# Editorial: Asbestos and disease genomics: is mesothelioma a genomic paradigm?

**DOI:** 10.3389/ftox.2024.1536344

**Published:** 2025-01-03

**Authors:** Marie-Claude Jaurand, Fiona Murphy, Emanuela Felley-Bosco

**Affiliations:** ^1^ Université de Paris, Centre de Recherche des Cordeliers, Inserm, Sorbonne Université, Functional Genomics of Solid Tumors, Paris, France; ^2^ Strathclyde Institute of Pharmacy and Biomedical Sciences, University of Strathclyde, Glasgow, United Kingdom; ^3^ Department of Biomedical Sciences, University of Lausanne, Lausanne, Switzerland

**Keywords:** asbestos, elongated mineral particles, mesothelioma, multi-omics-approaches, social impact, fiber pathogenicity paradigm

## 1 Introduction

A paradigm is a set of assumptions that creates a viewpoint of the world. However, as summarized by Carlo Rovelli (Carlo Rovelli, 2016), “scientific knowledge is the process of continuously modifying and improving our conception of the world, selectively and constantly questioning the assumptions on which it is based.” In this Editorial, we aim to challenge the current assumptions surrounding the concept of mesothelioma as a genomic disease through a comprehensive review of our understanding of disease pathogenesis and integration of recent findings reported in this Research Topic (Leinardi et al.; Sekido and Sato; Farahmand et al.; Fisher et al.).

The recognition of asbestos fibers as lung and pleural carcinogens has been long established from epidemiological data and is supported by experimental studies. The clear link between fiber inhalation and cancer raised important questions on the carcinogenic mechanism of action of asbestos fibers in exposed populations. Concomitantly, experimental studies of the pathological consequences of exposure to asbestos unraveled a role for specific features of fibers in disease pathogenesis. The integration of this knowledge is expressed in the fiber pathogenicity paradigm (FPP), which directly links the dimensions (diameter and length) and biopersistence of the fiber, in addition to dose, to mesothelioma hazard (Van Dorn, 2017; Rose, 2022). Advancing this work led to the formulation of a concept of “elongated mineral particles (EMPs)” to classify fiber types that share similar features with asbestos (Institute of Medicine, 2009). This concept triggered further studies to investigate the potential pathological potency of EMPs in relation to the asbestos hazard, especially at the thoracic level, and more recently flagged concerns over potential increasing exposure to novel engineered materials as a result of advances in (nano)technological developments of manufactured fibers (Nel, 2023).

## 2 Brief history

### 2.1 Discovery of asbestos-related diseases

Asbestos mining on an industrial scale started at the end of the 1800s, with the development of mesothelioma in exposed miners first reported 50 years later (Wagner et al., 1960). The use of asbestos has been regulated in many countries since 1995; however, several developed and developing countries continue to mine, export, and use asbestos in high volumes (Frank, 2020), with approximately 1,200,000 metric tons of asbestos used worldwide in 2021 (USGS, 2022 https://pubs.usgs.gov/publication/mcs2022). Over time, the risk of exposure identified at the workplace has been extended to using asbestos-containing material and during efforts to remove asbestos from existing structures (Gottesfeld, 2024). Secondary exposure scenarios have become an increasing cause for concern as many asbestos-containing buildings constructed in the mid-twentieth century are reaching their end of life and will require significant repair, reconstruction, or demolition in the near future. These existing and emerging threats have the potential to increase asbestos exposure in more diverse populations and continue the risk for the development of asbestos-related diseases (Alpert et al., 2020; Singh and Frank, 2023; Metintas et al., 2024).

Our historical experience with asbestos has left us with a heavy burden of disease. Lessons, however, have been learned that are relevant to both the scientific context, including an increased understanding of the mechanism underpinning fiber toxicity, and from an ethical perspective. Recognition of the long latency period from initial exposure to disease development and the lack of compensatory mechanisms within the body to neutralize pathogenic fibers highlights the need to address the humanitarian concern of ongoing exposure and anticipate health risks before developing new industrial procedures utilizing EMPs and during the design of novel fiber-like materials.

### 2.2 The remarkable characteristics of asbestos fibers related to toxicity

Experimental studies have revealed that fiber morphology and physico-chemical properties modulate the biological effects of asbestos fibers, emphasizing the role of fiber dimensions, especially length (Stanton et al., 1981). Additionally, *in vitro* acellular systems have been used to quantify the fiber dissolution rate and confirm the role of fiber durability in the biopersistence of pathogenic fibers. Epidemiological studies have substantiated these findings, especially on the relevance of fiber dimensions. From this research, it can be summarized that size, chemistry, and surface reactivity are basic parameters that govern toxicity. They are involved in biological responses such as fiber uptake and phagocytosis, interactions with biological molecules, genetic alterations, inflammation, immunity, translocation processes, and biopersistence (Sayan and Mossman, 2015; Nagai and Toyokuni, 2010; Donaldson et al., 2010; Kuroda, 2021; Huang et al., 2011). Further research has added several fiber parameters that are involved in the list of toxicological effects, such as rigidity. This information has been used to develop models to predict a mesothelioma potency hazard based on fiber dimensions (Nel, 2023; Wylie and Korchevskiy, 2023) or, with further refinement, the “fiber potential toxicity/pathogenicity index (FPTI),” which includes 18 parameters associated with an adverse effect in the pathological process (Gualtieri, 2018; Wylie and Korchevskiy, 2023).

The FPP is now being applied to assess the carcinogenic potential of fibrous particles such as glass and refractory fibers, carbon nanotubes (CNTs), metallic fibers, and new manufactured particles (high aspect ratio nanomaterials, or HARNs) (Nel, 2023; Murphy et al., 2021; Kane et al., 2018).

The FPP has left us with the legacy of continuing research into asbestos toxicity mechanisms and studying EMPs and HARNs to avoid health damage.

## 3 Mesothelial cell response to asbestos and mesothelioma characteristics

### 3.1 Early pleural responses to asbestos fibers

Experimental research has demonstrated a translocation of inhaled asbestos fibers toward the pleural space, although the mechanism is not fully understood (Miserocchi et al., 2008). The retention of asbestos fibers in the pleural cavity seems partly related to the size of stomata or pores through which pleural fluid drains to the lymphatic system (from 0.8 µm in mice to 10 µm in humans) (Schinwald et al., 2012). Subsequent accumulation of long fibers in the pleural cavity leads to direct irritation of the mesothelial layer, frustrated phagocytosis of pleural macrophages, and inflammation. Direct instillation of CNTs and other high-aspect ratio nanomaterials (HARN) into the pleural space have demonstrated a similar pathogenicity and mechanism of action to asbestos fibers in terms of production of oxidative stress, inflammation, and genotoxicity (Donaldson et al., 2013; Yoshida, 2019; Nagai and Toyokuni, 2010).

The mesothelial cell response to asbestos fibers was studied in cell culture models, including an SV40-immortalized, non-tumorigenic human mesothelial cell line. Normal mesothelial cells internalize the fibers, and chrysotile fibers were found in phagolysosomes with a lysosome degranulation (Nagai and Toyokuni, 2012). Inflammatory factors shown to be released *in vitro* by mesothelial cells may propagate a chronic inflammatory environment *in vivo,* subjecting mesothelial cells to ongoing oxidative stress, which may eventually result in cell transformation (Sayan and Mossman, 2015). In this Research Topic, Leinardi et al. provide a comprehensive review of the role inflammatory components released from cells after cell death can contribute to chronic disease development in the context of silica exposure. While silica induces disease by the release of pro-inflammatory damage-associated molecular patterns, including HMGB1 from macrophages, asbestos carcinogenesis can be promoted by the release of HMGB1 directly from mesothelial cells (Suarez et al., 2023).

To examine early changes along the mesothelium in response to fibers, a transcriptomic kinetic analysis of mesothelial cells exposed by injection of fibers into the pleural cavity of C57BL/6 mice was carried out at timepoints between 1 week up to 20 months after exposure (Chernova et al., 2017). Samples consisted of long and straight CNTs, short CNTs, and long (carcinogenic) and short (lower pathogenicity) amosite asbestos fibers (Chernova et al., 2017). Inflammatory lesions studied from 1 week to 6 months after injection were similar in mice exposed to both long samples in terms of cell components in the pleural cavity and expression of inflammatory response genes, whereas gene expression from mice exposed to short fibers and controls clustered together. Inflammatory response pathways and activation of kinases involved in pro-oncogenic pathways were identified in early lesions with dysregulation maintained through to tumor development. The status of the *Cdkn2a* gene (encoding two proteins, p16^Ink4a^ and p19^ARF^), an ortholog of the key tumor suppressor genes (TSGs) in human mesothelioma *CDKN2A* (encoding *P16* ^*INK4a*^ and *P14* ^AR*F*^), was examined in inflammatory lesions at 1-year post injection before tumors developed and in tumors induced by exposure to long CNT and amosite asbestos (Chernova et al., 2017). The authors reported silencing of *Cdkn2a* (*Ink4a/Arf*) by hypermethylation and co-deletion of the proteins in the fiber-induced inflammatory lesions that increased in tumors, which also acquired a *p19/Arf* deletion. This shows that epigenetic changes are present early in an inflammatory, pre-tumoral stage and emphasizes the similarities with human pleural mesothelioma (PM) (Chernova et al., 2017).

Transcriptome analyses of asbestos-induced inflamed tissue were investigated in heterozygous *Nƒ2* ^+/−^C57Bl6 mice intraperitoneally exposed to crocidolite fibers for 12 weeks and assessed up to 33 weeks after the last exposure (Rehrauer et al., 2018). They revealed a decreased level of *Nƒ2* expression and activation of Yap/Taz localized in the cell nucleus in inflamed mesothelium, which increased in tumors, indicating deregulation of the Hippo pathway in these mice (Rehrauer et al., 2018). Although conducted in a genetically modified mouse model to increase mesothelioma susceptibility, this study highlights the potential role that dysregulation of the Hippo pathway due to *Nf2* mutation plays in the progression of mesothelioma. Although *Nf2* loss is regularly identified in mesothelioma tumors reviewed in the article by Sekido and Sato as part of this Research Topic, in human disease, the *NF2* mutation appears to be a late event, indicating that genomic damage of *NF2* may not be a direct asbestos effect but a result of the chronic inflammatory and oxidative environment.

### 3.2 Histopathology and molecular genetic characterization of PM

The characteristics of human PM are continuously evolving and concern several fields of cell and tumor biology, from histological classification, genetic, epigenetic, and chromosomal status, state of regulatory pathways, and cell-to-cell interactions with the immunological microenvironment.

The recent histological classification of PM retains the three main histologic subtypes: epithelioid, biphasic, and sarcomatoid, with biphasic including epithelioid and sarcomatoid elements. Mesothelioma subtypes show a variety of architectures, cellular aspects, and stroma, and their prognosis is different, with a worse survival for the sarcomatoid subtype than the epithelioid (Sauter et al., 2022; Husain et al., 2024). There was no epidemiological evidence of an association between the histological classification of mesothelioma and exposure to a given type of asbestos fibers (Vorster et al., 2022; Franklin et al., 2016). However, in a genetically engineered conditional mouse model, where mostly sarcomatoid mesothelioma develops spontaneously after co-deletion of *Nf2*, *p53*, and *Cdkn2a* in mesothelial cells, asbestos exposure accelerates the onset of mostly epithelioid tumors (Farahmand et al.). One notable feature of the response to asbestos exposure in this genetically modified mouse model is the increased recruitment of macrophages observed as a precursor to mesothelioma development. This raises the possibility that the tumor microenvironment and epigenetic events downstream of asbestos exposure provide cues favoring proliferation when compared to the tumor developing in the absence of asbestos. This is also supported by the observation that different methylation levels of CpG sites were detected within tumors and were reflective of intratumor heterogeneity of the histological subtype. DNA methylation was preferentially located in CpG islands for the sarcomatoid subtype while mainly located in non-CpG islands for the areas with high epithelioid histology, suggesting that histo-molecular gradients are linked to epigenetic regulation (Blum et al., 2019).

While there are limited studies showing the effects of asbestos fibers on the regulation of gene expression after short-term exposure of mesothelial cells to asbestos fibers, there is currently a large body of data on the pathological and biomolecular characteristics of PM. They are usually investigated long after the onset of the tumors, which are biopsied a long time after the beginning of exposure, possibly several decades.

Investigations of the molecular landscape of mesothelioma began in the 2000s with the development of methodologies for large-scale analytical methods that provide high-throughput analysis of biological data. The genetic and epigenetic modifications of the tumors were studied using multi-omic approaches, such as next-generation sequencing and microarrays. As observed in histology, PM is a heterogeneous tumor, with a rather low number of somatic gene mutations compared to other cancers, but with a high number and types of chromosomal rearrangements, copy number alterations, genome duplication, and mutations in a number of key genes, most of them being TSGs (*CDKN2A*, *BAP1*, *NF2*, *SETD2*, *LATS2*, and *TP53*) and a mutation in the *TERT* promoter (Bueno et al., 2016; Meiller et al., 2021; Febres-Aldana et al., 2024; Creaney et al., 2022; Nair et al., 2023; Mangiante et al., 2023). At a lower rate, mutations were detected in genes of the SWI/SNF family (*ARID1A*, *ARID2*, and *SMARCA4*) and genes related to histone methylation (*KMT2D* and *SETD2*) were mutually exclusive (Quetel et al., 2020; Febres-Aldana et al., 2024).

Transcriptomic analyses revealed that the heterogeneous molecular profiles of PM could be identified allowing a molecular classification of pleural PM. This refined classification identified several subgroups characterized by different molecular profiles and gene alterations that distinguish the epithelioid from the sarcomatoid phenotype and were linked to the patients’ survival, with a better outcome for epithelioid molecular profile than sarcomatoid (Bueno et al., 2016; Blum et al., 2019). Investigation of the intra-tumor heterogeneity showed that, in reality, tumors from individual patients are composed of a combination of epithelioid-like and sarcomatoid-like components (defined by E/S score) (Blum et al., 2019; Alcala et al., 2019). There was a significant enrichment of *BAP1* and *SETD2* mutations in tumors of the highest E score, of *TERT*_prom, *NF2*, and *TP53* alterations in tumors with the highest S score, and *LATS2* was more frequently altered in nonepithelioid PM and positively associated with the S score (Quetel et al., 2020; Blum et al., 2019).

A genetic predisposition was suggested in families of mesothelioma cases, and a high incidence of mesothelioma related to *BAP1* tumor predisposition syndrome multifunctional gene (BRCA1-associated protein, *BAP-1* gene) was discovered (Testa et al., 2011)**.** Other studies reported a significant proportion of frequency of germline mutations and most pathogenic variants in DNA repair and TSGs (Panou et al., 2018; Belcaid et al., 2023).

Additional evidence for the influence of genetic predisposition in mesothelioma pathogenesis is suggested by experimental models, such as the Cross Collaborative MexTAg mouse model, where 72 different genetic backgrounds were tested, resulting in the identification of genetic variants predictive of different disease latency (Fisher et al.).

Knowing that asbestos induces inflammation and chromosome damage, including chromosome missegregation in mesothelial and other mammalian cells, research was performed to detect mutation signatures in PM (Huang et al., 2011). Recently, an analysis of clinical genomic profiling of patients with PM identified near-haploidization in an aggressive biphasic subtype that occurred in younger patients without asbestos exposure (Yang et al., 2024)**.** Losses in chromosomes 14q have been reported in a few studies; for example, recurrent loss in 14q11.2-q21 was found in asbestos-exposed patients compared to patients not exposed, losses in chromosome 14q were similarly identified in fiber-induced murine mesothelioma (Björkqvist et al., 1999; Jean et al., 2011). While DNA oxidation was reported in asbestos-exposed animals, no reactive oxygen species (ROS)-induced mutation signature was reported in human PM. Recently, data on PM were reinvestigated with a new statistical analysis, signature variability analysis (SVA), which considers the heterogeneity of the tumor mutations and the variability of mutations within and across tumors (Morrison et al., 2023). While there was no difference in copy number alteration and single base substitution signatures between exposed and unexposed patients, tumors from patients exposed to asbestos have more within-sample signature diversity and less across-sample heterogeneity than those from unexposed patients, suggesting that SVA could be used to generate a footprint of asbestos exposure. Interestingly, analysis of biopsies taken at distinct anatomical sites revealed intra-tumor heterogeneity (Meiller et al., 2021; Zhang et al., 2021). In this study, NGS performed on key mesothelioma genes showed heterogenous variants, especially *NF2*, which appears to be a late event.

A recent meta-analysis of DNA methylation in PM investigated 53 studies for DNA methylation of genes in mesothelioma in a total of 97 genes, including microRNAs (miRNA) analyzed at CpG methylation sites (Vandenhoeck et al., 2021). In this study, methylation was more frequent in mesotheliomas of the epithelioid subtype. The number of aberrantly methylated genes was also positively linked to asbestos body counts in the lung, which is a signature of asbestos exposure. The genes most significantly hypermethylated in mesothelioma in comparison with normal tissue are *CDH1*, *ESR1*, miR-34b/c, *PGR*, *RARb*, *SFRP1*, and *WIF1*, and one, *APC* is hypomethylated.

A promoter hypermethylation of the cell cycle control-associated genes *CDKN2A*, *CDKN2B*, *RASSF1*, *CCND2*, *APC*, and *HPPBP1* was found in asbestos-exposed patients with a high asbestos body burden after control of confounding factors (Christensen et al., 2008). Asbestos body counts were also positively linked to methylation at CpG sites (Christensen et al., 2009).

Dysregulation of both miRNA and long non-coding RNAs (LncRNAs) has been identified in mesothelioma tumors (Abd-Elmawla et al., 2023; Xu et al., 2023). Many miRNAs target epithelial or mesenchymal markers, and their expression is dependent on the E/S score (Blum et al., 2019). LncRNA, *such as NEAT1, PCT6, HOTAIR,* and *GAS5*, were identified as potential biomarkers.

RNA-editing patterns of PM have been studied in humans according to the E/S scores of the tumors in untranslated regions (UTR) of transcripts and in introns. Results showed that PM with a high E-score had RNA frequency editing at the 3′UTR and in introns in PM with a high S score (Felley-Bosco et al., 2023). Then, the regions vary with the EMT, consistent with the epigenetic regulation of EMT. In asbestos-exposed *Nf2* ^+/−^ mice, an RNA-editing signature, mediated by adenosine-deaminase-acting-on dsRNA (ADAR), was found in inflamed tissues 33 weeks after exposure, with a higher number of RNA-editing events in tumors (Rehrauer et al., 2018).

Research on the PM microenvironment aims to identify the different cell components, including immune cells, with the goal of reactivating the immune defense. Single-cell transcriptomics identified a sarcomatoid-enriched phenotype associated with fetal-like endothelial cells, CXCL9/10/11+ macrophages, and cytotoxic, regulatory, and exhausted T lymphocytes (Giotti B, et al., 2024), in line with bulk-RNA studies (Alcala et al., 2019; Mangiante et al., 2023). Detailed analysis of tumor cell populations will permit novel immunotherapy to increase mesothelioma cell susceptibility to death via immune cell activity.

## 4 Conclusion

Research on PM concerning the role and mechanism of action of asbestos fibers has demonstrated the role of asbestos as a major etiological factor and its multifactorial mechanism of action.

Asbestos specificities are related to the mechanism of fiber–cell interaction (phagocytosis) and genomic damage. One of the key mechanisms of cancer involves gene mutations, which are not at a high level in PM, but chromosomal damage is significant. As PM is an infrequent cancer among the whole population, largely dependent on asbestos exposure in diverse populations, it is likely that DNA repair polymorphism plays a significant role in PM induction. Additionally, a background of persistent inflammation can act at different levels (ROS production, increased proliferation of pre-malignant cells, immunosuppression) to elicit the neoplastic progression and modify the tumor microenvironment. The different tumor microenvironments according to PM histology and the E and S components of the tumor are remarkable, in line with tumoral/EMT evolution, posing a significant challenge for effective therapeutic intervention.

While mesothelioma genomics provided us with footprints on the link between established disease and asbestos, early imprints are poorly identified. Investigations of the early effects of asbestos on mesothelial cells and pleura have shown activation of inflammatory pathways and apoptosis and permitted identifying early genetic and epigenetic impacts at the onset of exposure (Sayan and Mossman, 2015; Huang et al., 2011; Chernova et al., 2017; Rehrauer et al., 2018). Studies of asbestos-exposed mice heterozygous on key TSGs confirmed the importance of these genes in the neoplastic progression of mesothelial cells under asbestos exposure [reviewed in Blanquart et al. (2020) and Testa and Berns (2020)]. Although *NF2* is frequently altered, its alteration is a late event (reviewed in Sekido and Sato), and the application of novel technologies will reveal cell environment cues driving that alteration.

Presently, we may propose that PM appears as a double paradigm, toxicologic for approaches to further research on EMPs and genomic for asbestos diseases due to some specific molecular changes. Nevertheless, progress must be made to distinguish the asbestos mechanism of action from the neoplastic progression of mesothelial cells.
